# Integrating Dietary Impacts in Food Life Cycle Assessment

**DOI:** 10.3389/fnut.2022.898180

**Published:** 2022-07-13

**Authors:** Olivier Jolliet

**Affiliations:** ^1^Department of Environmental Health Sciences, School of Public Health, University of Michigan, Ann Arbor, MI, United States; ^2^Quantitative Sustainability Assessment, Department of Environmental and Resource Engineering, Technical University of Denmark, Kongens Lyngby, Denmark

**Keywords:** food, life cycle assessment, sustainable, nutrition, health nutrient index, human health

## Abstract

Food production and food consumption have been too long studied separately. This paper therefore reviews progresses in assessment methods and identifies how nutrition effects on human health and environmental impacts of the entire food production and consumption can and should be consistently and systematically assessed, on a life cycle-based and a health-based perspective. Main observations include: (a) The strong activity in the Life Cycle Assessment (LCA) of a large range of agriculture production, covering beyond carbon footprint the biodiversity and health impacts of land, water, fertilizers, and pesticide use. (b) The multi-functionality of all foods and the need to compare a wide range of possible alternative including comparing serving size, meal alternatives and diets. (c) The availability of epidemiological dietary risk factors expressed in DALYs, enabling the creation of an additional LCA nutritional impact category and providing much broader flexibility in the choice of the functional unit and the kind of valid comparison LCA can address. (d) The need to use Big Data and machine learning method to better understand interactions and propose healthy and sustainable food baskets. As illustrated by the fruit yogurt example, dietary impacts on human health often dominate the life cycle impacts on human health and it is strongly recommended to consider them in the life cycle inventory and impact assessment of all commodities and foods that will eventually be consumed.

## Introduction

Food production and consumption are key factors both for our environment and for our health (1). Sustainable production and processing of food is a crucial question in a time where eutrophication, particulate matter, water, and land use from food production exceeds planetary boundaries, set high pressure on our climate, and is a high factor responsible for the threat on hundred thousands of endangered species. What “offerings of food” can be produced and how the entire world population can be fed, while limiting environmental impact and maintaining these within the limits of the planetary boundary are key challenges for our societies (2).

Food and diet are also key determinants of health. Most of the dominant risk factors identified by the Global Burden of Disease (GBD)–(3) are directly associated with dietary risks, or indirectly related to nutrition (e.g., high systolic blood pressure, low-density cholesterol, plasma glucose, and body mass index), and are responsible for tens of millions of death annually (4).^1^ A main challenge is the multidimensional nature of diet-health interactions, in term of the multiplicity of foods, health outcomes and their possible combinations which makes it difficult for the consumer to identify what really matters.

Major scientific progress have been achieved in the last three decades in assessing agriculture and food production over life cycle showing the importance of direct emission on field, as well as the high burden associated with food waste. Thoma et al. (5) provide a very informative overview of how Life Cycle Assessment (LCA) can be applied to agriculture and food production, for each of the four main LCA phases, i.e., (1) the goal and scope definition that determine the functional unit retained as the basis for the comparison as well as the food system boundaries, (2) an inventory of flows coming from the environment or released to the environment per defined functional unit, (3) the associated impact on human health, ecosystem biodiversity and resource use, determined using so called “midpoint category” (e.g., fine particulate, human toxicity, or land use and eutrophication) that provide specific characterization to each pathway, and (4) the interpretation phase that interprets the different results of each phase and assesses uncertainties. However, food production and food consumption have been too long studied separately (6), and both nutrition and environmental fields have often drawn conclusions without accounting for the complex interactions between these dimensions. It is especially strange that food LCAs, aiming to cover holistically the whole life cycle of food systems, have in practice mostly neglected the dominant dietary impacts of food on human health during use stage. Also LCA usually compares foods based on a single functional unit, which might fail to fully reflect the natural multi-functionality of foods and diets (7, 8).

This paper therefore reviews progresses in the environmental life cycle assessment of agriculture and food products. It then identifies how nutrition effects on human health and environmental impacts of the entire food production and consumption during use stage can be consistently assessed, both on a life cycle-based and a health-based perspective. It also illustrates how this opens new possibilities to compare a broader range of food alternatives and account for the multiple nutrients and functionalities of food.

## State of the Art and Progress in Life Cycle Assessment of Agriculture and Food Systems

Major progresses have been achieved in assessing the environmental performances of agriculture production and food systems over life cycle. Food is one of the domain with most LCA activity since the early 1990s, and has contributed to pioneer LCA methodological approaches, with several milestones and unique developments. From 1993 to 1996 a European concerted action on harmonization of environmental LCA for agriculture determined and compared six evaluations of impacts of wheat production, also setting the basis for the ISO hierarchy on allocation (9). Intensive developments of Process-oriented life cycle inventory databases have led to the creation of several food oriented databases covering the entire supply chain of agriculture production, including the World Food LCA Database and its integration in ecoinvent (10), the Agribalyse Database (11), or the Agri-footprint database (12). The emergence of Multi-Regional Input-Output databases and their combination with always more comprehensive global Life Cycle Impact Assessment method enable us to account for the global nature of food production (13) and the trade-off between local production [Poore and Nemecek (14) for country specific production inventories] and transported food produced in best suited climatic condition and location (15).

Food losses have been modeled in further details and identified as a major driver of environmental impacts of foods, with high potential for improvement (16, 17). Food processing has been included in several LCA studies [e.g., Kim et al. (18) for cheese], but this is certainly a domain together with the cooking mode that would deserve additional attention, data collection, and further development. This is in particular true for pre-processed mixed dishes and meals that become increasingly popular.

Major advances have also been achieved for the Life Cycle Impact Assessment (LCIA) of food-related impacts on human health and on ecosystem quality. Eutrophication fate and effect factors have been developed (19) and further refined in the frame of the UN project for developing a consensus-based Global Impact Assessment Method (GLAM)–(20, 21). Impacts of pesticides on ecosystem quality and human health are increasingly characterized (22, 23), also accounting for the pesticide residues consumed in multiple crop types (24, 25). Main impacts on human health associated with the creation of secondary fine particulate smaller than 2.5 μm diameter (PM_2.5_) have been modeled in detail, with agriculture specific characterization factors expressed in e.g., DALY/kg_precursor_ in particular for ammonia emissions (26). For land use and land use change impacts on biodiversity, major progress have been reached by Chaudhary et al. (27) and Chaudhary and Brooks (28) to assess ecoregion specific impacts for five different types of land use. Kuipers et al. (29) provided additional information on habitat fragmentation and global extinction probabilities. For water footprint, the impact of agriculture as the dominant consumptive user of water can now be assessed using the AWARE method (30) enabling to better assess the depletion of water use for both ecosystems and humans. In a study of the water footprint of US dairy milk in 50 states and 18 water basins, Henderson et al. (31) demonstrate the very localized character of water impacts for feed and dairy production. For assessing the carbon footprint, LCIA methods such as Impact World+ (32) enable to calculate impacts for different time periods (both for the first 100 years and for longer term), avoiding the arbitrary choice of a fixed time horizon, which could strongly influence the impacts of methane relative to CO2.

**FIGURE 1:**
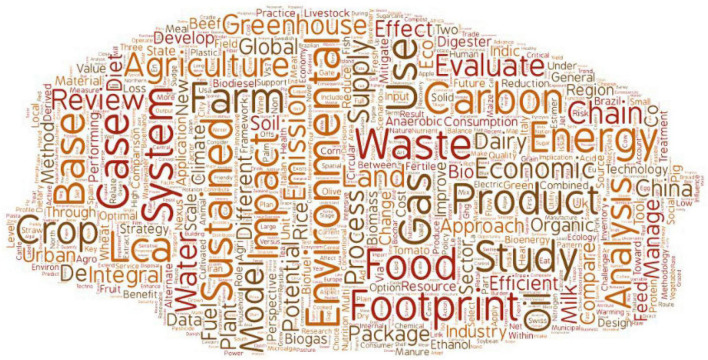
Word cloud of most frequent words in the titles of 4,131 papers containing life cycle assessment together with agriculture or food in their title, abstract or keywords. Created with WordArt and Scopus on 1 March 2022.

The LCA food conference (33), hold every other years, has provided since the nineties a forum for intensive exchange on data, methods and the assessment of multiple crops and diets. This intense activity in agriculture and food LCAs is reflected in the more than 4,000 papers that have Life Cycle Assessment together with Agriculture or Food in their title, abstract or keywords. Figure 1 presents the most frequent words in the titles of these papers. Interestingly for fields of study, Waste is as prominent as Food, Agriculture or Crop. In terms of environmental impacts as expected Carbon Footprint, Greenhouse Gases are often mentioned together with Water issues. Dairy and Milk are the most prominent commodities, followed by Beef, Rice, Pig, Tomato with also strong occurrence of Energy related production with Oil, Bioethanol, Biodiesel, or Biogas. For regions, studies on China are most frequent, followed by Brazil, United Kingdom, Iran, Europe/EU, and Switzerland.

## The Multi-Functional Nature of Foods—Beyond Functional Unit

**FIGURE 2:**
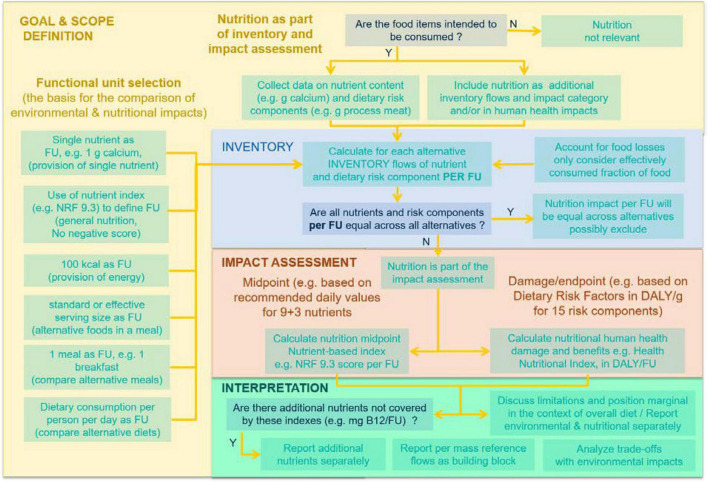
Integration of the nutrition impact associated with food consumption during use stage, in the life cycle assessment of foods or food ingredients.

In LCA an important choice is the selection of the functional unit (FU), i.e., the basis for comparing different scenarios, all emissions and impacts across alternatives being calculated per functional unit. For agriculture of food, a wide range of functional units have been used, including per kg or 100 g, per kcal, per serving, per meal or per person per day for an entire diet (Figure 2, left column). LCA studies only use a single metric at a time as functional unit, whereas multi functionality is intrinsic to food. This becomes a problem if it is assumed unrealistically that all functions and benefits of food can be reduced to a single variable, that accounts for the entire nutritional function for all compared alternatives. In contrast, a unique functional unit is not a problem if the environmental or health aspects that are not covered by the functional unit are included in the impact assessment. Rather than debating whether nutrition should be considered within the functional unit of a LCA or within the impact assessment (8), we propose to use both approaches, applying the following recommendations, first for the functional unit:

a) It is useful to systematically reporting of mass-based results and mass is a main reference flow, but mass is very rarely a good functional unit, and is most of the time misleading, since substitution and alternatives is not primarily mass related.
b) The serving size has been created by food agencies and is used worldwide as a default basis for comparison, and could be one interesting functional unit, but needs to be complemented by a health/nutritional impact assessment. Other functional units could also be legitimately considered such as kcal if the function is to provide energy, etc.
c) The functionality of food is very rarely mono-dimensional or mono-functional. To believe that we can force the multi-functionality of foods into a common functional unit that fully reflects health performances and nutrition and the function of nutrition [more than 10,000 different nutrients in one food—(34)] is an illusion. It is trying to force reality to fit a too simplified LCA framework.
d) Since most nutrient index include detrimental nutrients (e.g., sodium), the use of nutrient index score as a functional unit is debatable, since it indirectly qualifies detrimental components as part of the food function. It becomes even impossible to compare alternatives if some of the net nutrient scores are negative (more detrimental than beneficial nutrients). These detrimental impacts rather belong in LCA to the impact assessment phase.
e) The nutritional and human health impacts performances per functional unit of two food items, two breakfasts, two meals, or two diets, are in general different (even very different sometimes). These differences in human health impacts or benefits can and should be accounted for separately in the impact assessment, considering both impacts and benefits. The assessment of human health impacts (with uncertainty) of nutrition needs therefore to be considered in food LCAs, unless they are exactly equal per FU.
f) In the rare cases where compared alternatives have exactly equal human health impacts related to the food nutritional value, this is not a problem and there is no double counting since the human health impacts are equal and do not bias the comparison between these alternatives. In addition, it is still always useful to put in perspective and compare the human health impacts of food production with the often dominant human health impacts of their consumption.

Best practice recommendations were further developed under the FAO umbrella to address the intended purpose of an LCA study and related modeling approach, choice of an appropriate functional unit, assessment of nutritional value, and reporting nutritional LCA results (35). Main recommendations included: (a) When nutrients are and/or nutrition is relevant to the decision-maker and decision context, nutrient and nutrition related impacts should be considered in the LCA; (b) as many essential nutrients as possible should be reported in the inventory; (c) though research on the potential human health impacts of food items is at an early stage, it is recommended using in the life cycle impact assessment phase a nutrition impact category to account for the benefits or impacts of nutrition on human health. In this line of thought, having a human health dietary impact category in LCIA, provides much more flexibility for comparing a wide variety of foods in a consistent way. The next section will review how this can be achieved.

## Consistently Accounting for Dietary Impacts on Human Health in Life Cycle Assessment

How can dietary impacts during use stage be assessed in LCA, ensuring consistency with other types of human health impacts considered during production stage (such as the health impacts associated with the generation of fine particulate)?

Epidemiologically-determined risk ratios from e.g., the GBD have become available for various nutrients and food groups (3, 4). According to the GBD, beneficial risk components include fluid milk, nuts and seeds, fruits, calcium (excluded for fluid milk to avoid double counting), omega-3 fatty acids from seafood, fibers (fibers from fruits, vegetables, legumes, and whole grains differentiated from other sources), and polyunsaturated fatty acids (PUFAs). GBD detrimental factors include health damages associated with processed meat, red meat, trans fatty acids (TFAs), sugar-sweetened beverages (SSBs, mediated through body mass index) and sodium (mediated through blood pressure). For LCA, depending on the LCA scope definition, there is a need to look both at overall diet changes, and analyze marginal changes in the context of an overall diet.

Figure 2 summarizes how these relative risks, burden rates and exposures from the GBD can be used within LCA for marginal effects of individual food items. We collect data on the nutrient content and food group components of each of the food ingredient (e.g., g calcium or g fruits per 100 g of milk, strawberries and corn syrup for a strawberry yogurt). Based on the food composition, we first determine the content levels to each risk factors by quantifying as inventory flows the amount of dietary risk component r per functional unit [*d*_*r*_, in e.g., g calcium or g fruits per serving of yogurt, Table 1; also see Fulgoni et al. (36)]. These amounts are then multiplied by the impact assessment characterization factor, the so-called Dietary Risk Factor expressed in μDALY per g of each risk components (*DRF*_*r*_, in e.g., μDALY/g_calcium_ or g_fruit_) and summed up across all relevant risk factors to yield the impact per functional unit (e.g., in μDALY/serving of yogurt), or the Health Nutrient Index (HENI) score expressed in minutes of healthy life gained per serving, considering that there are 0.526 million seconds in a year (37):


$$H\,E\,N\,I=-0.526\times \sum_{r=1}^{n}\left(d_{r}\times D\,R\,F_{r}\right)$$

**TABLE 1:** Calculation of the health nutrient index for a three ingredients strawberry yogurt [adapted from Thoma et al. (5)].

Yogurt composition	Mass	Energy	Fibers fruits	Calcium	PUFA	Fruit	Sodium	Transfat	
	[g/serving]	[kcal/serving]	[g/serving]	[g/serving]	[g/serving]	[g/serving]	[g/serving]	[g/serving]	
Corn syrup	10.0	28.4					0.006	0.003	
Strawberries	6.0	1.9	0.12	0.00	0.01	5.95			
Yogurt plain low fat milk	154.0	97.1		0.28	0.07		0.108	0.027	
**Total d_*r*_ strawberry yogurt**	**170.0**	**127.4**	**0.12**	**0.28**	**0.08**	**5.95**	**0.114**	**0.030**	
**Dietary risk factor *DRF*_*r*_**	[μDALY/g]		–0.18	–5.15	–0.61	–0.19	13.90	4.44	**Total**
**Health nutrient index, HENI**	[min gained/serving]		–0.01	–0.77	–0.02	–0.59	0.83	0.07	**–0.48**

*Bold are totals.*

Stylianou et al. (37, Supplementary Table S3) determined DRF values for the US for the 16 GBD dietary risk factors, accounting for 400 risk-outcome associations, stratified by 15 age groups and gender. Combining these with food composition and food consumption data enabled them to determine the minutes of healthy life lost or gained per 100 g, per kcal and per serving for more than 5,800 foods items consumed in the United States. Since some essential nutrients are not specifically covered by the GBD (e.g., anti-oxidants, Vitamin B12, or other essential vitamins), complementary nutrient might be considered at midpoint level, to complement the HENI score.

For 170 g serving of the strawberry yogurt given as example in Table 1, this calculation of the HENI score results in a reduction of –0.92 μDALY/serving, i.e., 0.5 min of healthy life gained per serving, considering that there are 0.526 million seconds in a year. This is the difference between 1.5 min gained mostly via calcium (since yogurt is not considered as milk) and fruit, minus 0.9 min mainly associated with sodium. As further discussed by Thoma et al. (5), these dietary impacts of 0.5 min per serving yogurt are restricted compared to other foods such as hot dogs (36 min lost per hot dog), but are still an order of magnitude higher than the estimate of climate change and fine particulate, thus the importance to account for them.

The marginal human health impacts of individual food items should be considered in the context of an overall diet. This is accounted for by the GBD maximum theoretical limits (TMRELs) above or below which there is no benefit or impact as defined by the Global Burden of Disease [see, for example, Supplementary Table S3 of Stylianou et al. (37)], whereas the majority of the population is in the active range of consumption for which marginal changes leads to effective changes. For more substantial dietary changes, a multiplicative approach should be used according to the GBD, as applied for entire diets by Walker et al. (38).

## Discussion, Conclusion, and Perspectives

The proposed approach enables us to consistently account for dietary impacts, in parallel to environmental impacts, a major progress in determining the life cycle impacts of foods on human health, with the possibility to consider country specific mortality and morbidity rates. As illustrated in Figure 2, as soon as an LCA of agriculture and food systems address commodities that are intended to be consumed, or are ingredients of foods to be consumed, it is strongly recommended to consider their nutritional impacts in the life cycle inventory and impact assessment. Since these dietary impacts on health are expressed in DALYs (detrimental effects) or avoided DALYS (beneficial effects), they can directly be compared and summed up at damage level with the other impacts such as human toxicity, impacts of pesticides residues (24) and fine particulate damages on human health, while keeping track of the respective contributions.

Several limitations need to be further addressed: The present resolution of epidemiological data is still course, with e.g., all fruits considered as equally beneficial per g. The underlying data are usually analyzed for one or two risk factors at a time and do not fully reflect the combined interactions and confounding factors between substitutions, since increasing a serving size of a food group also results in decreasing the consumption of other food items. There is still ongoing debate for multiple items [see e.g., Stylianou et al. (39) for milk that could have additional detrimental effects on prostate cancer or and beneficial effects for stoke according to various meta studies] and regular revisions in the expert judgment of the GBD author. So far the HENI index has strictly followed the GBD risk estimates, but there is also a need to further stabilize the GBD data that have been changing substantially with time. For example, between the 2016 GBD data used in Stylianou et al. (37) and the 2019 GBD data (3) relative risks for red meat have been multiplied by a factor 5, and reduced by a factor 10 for the benefits of omega-3 acids in seafood.

The rapidly growing realm of data and of machine learning techniques made available offers interesting perspectives to address these limitations. Beyond the GBD data, the merging of different databases offers very interesting perspectives on health analysis. A good example is the combination of the NHANES effort that includes data on nutrition, physical activity, occupation, metabolism, and measured chemical biomarkers and biomarkers of physiological indicators, with mortality data on each of the participants. Zhao et al. (40) used applied survival random forest (41) to 47,000 individuals of this cohort to analyze the combined effect and respective importance of physiological indicators on all-cause mortality. This will enable us in the near future to identify more advanced dose-responses and quantify multi-stressor risks in an exposome-based approach that can look at the combined effects of e.g., nutrition, pesticide residues and physical activity on mortality.

## Author Contributions

The author confirms being the sole contributor of this work and has approved it for publication.

## Conflict of Interest

OJ is an independent member of the Sustainable Nutrition Scientific Board supported by Nutella.

## Publisher’s Note

All claims expressed in this article are solely those of the authors and do not necessarily represent those of their affiliated organizations, or those of the publisher, the editors and the reviewers. Any product that may be evaluated in this article, or claim that may be made by its manufacturer, is not guaranteed or endorsed by the publisher.

## Footnotes

1
https://vizhub.healthdata.org/gbd-compare/
